# Transcriptome and anatomical analysis of *Stipa breviflora* in response to different grazing intensities in desert steppe

**DOI:** 10.3389/fpls.2024.1414093

**Published:** 2024-06-10

**Authors:** Xiaoyu Wang, Jierui Wu, Rui Dai, Zhiqiang Zhang, Yunbo Wang, Fugui Mi

**Affiliations:** Key Laboratory of Grassland Resources of the Ministry of Education, College of Grassland, Resources and Environment, Inner Mongolia Agricultural University, Hohhot, China

**Keywords:** *Stipa breviflora*, grazing intensity, transcriptome, gene expression level, enzyme activities

## Abstract

*Stipa breviflora* is a dominant species in the desert steppe of Northern China. Grazing is the main land use pattern of grassland, which could cause a variety of adaptive evolutionary mechanisms in plant community composition as well as individual plant growth and morphological characteristics. However, very little is known about the morphological structure and transcriptional regulation response to different grazing intensities in *S. breviflora*. In this study, transcriptome and anatomical analyses of *S. breviflora* under different grazing intensities, including no grazing, moderate grazing, and heavy grazing, were performed. The anatomical analysis results showed that epidermis cells and xylems significantly thicken with grazing intensity, suggesting that grazing results in increasing lignification. Furthermore, the components of cell walls such as lignin, cellulose, hemicellulose, and pectin were all increased dramatically and significantly under both moderate and heavy grazing. Transcriptome analysis showed that the differentially expressed genes related to different grazing intensities were also engaged in plant cell wall formation and in photosynthesis and respiration. In addition, the activities of ATP synthase and Rubisco-activating enzyme increased significantly with enhanced grazing intensity and differed significantly between moderate and heavy grazing intensities. The trends in transcriptome and plant phenotype changes are consistent. Taken together, these results indicated that *S. breviflora* has evolved a grazing tolerance strategy under long-term grazing conditions, influencing photosynthesis and respiration in terms of its own structure and enzyme activities in the body, to maintain normal life activities under different grazing conditions.

## 1 Introduction

Grasslands cover approximately 40% of the global land surface (Hufkens et al., 2016; He et al., 2020), which are one of the multi-functional terrestrial ecosystems that play a key role in providing multiple ecosystem services (Eldridge & Delgado-Baquerizo, 2017; Yan et al., 2020). It is also essential for water and soil conservation and protection, climate regulation, and biodiversity maintenance (Costanza et al., 1997; Nearing et al., 2005; Fan et al., 2019). In recent years, there have been increasing demands on grasslands due to rapid economic development (Kemp et al., 2013; Fetzel et al., 2017).

The main land use pattern of grasslands is grazing (Li et al., 2015). However, some changes and successions of grasslands are caused by grazing, which influences the function and structure of grassland ecosystems more deeply, especially the multiple plant characteristics and soil properties of grasslands (Milchunas et al., 1988; Liu C. et al., 2021; McNaughton, 1983; Sun et al., 2020; Zhou et al., 2017). Different grazing intensities lead to significant changes in plant community composition, aboveground biomass, and soil physicochemical properties (Liu M. et al., 2021). Generally, light or moderate grazing can improve grassland diversity and productivity, while heavy grazing leads to serious grassland degradation (Kemp et al., 2013; McNaughton, 1979). Degradation of grasslands due to overgrazing has emerged as a significant global ecological issue (Dlamini et al., 2016; Eldridge et al., 2017; van de Koppel et al., 1997; Yang et al., 2023). Long-term grazing caused a variety of adaptive evolutionary mechanisms in individual plant growth and morphological characteristics such as plant height (Liu et al., 2022), abundance (Ridding et al., 2021), and biomass (Hao and He, 2019). It has been shown that plateau plants adapt to grazing stress by increasing the thickness of their leaves, cuticles, and phloem. The mesophyll cell area, as well as the stem epidermal cell area of *Poa alpigena*, decreased in response to minor variations in grazing intensity, but overgrazing did not change its density. However, overgrazing induced a shortening of the leaves and stems, indicating that overgrazing has a dwarfing effect on *Poa alpigena* (Shi et al., 2022). Long-term grazing can even affect the plasticity of gene expression and affect plant physiological and metabolic pathways (Dang et al., 2021).

*Stipa breviflora* (Poaceae) is a dominant perennial grass that is widely distributed in desert steppe regions like the western Inner Mongolia (Ren et al., 2017). It has attracted attention for its excellent palatability, grazing tolerance, and drought resistance. Because of the advantageous characteristics of this plant species, it is also employed for restoring degraded grasslands (Zhang et al., 2012). As a dominant species in desert grasslands, *S. breviflora* performs differently under different grazing intensities. A previous study showed that the aboveground and litter biomass of *S. breviflora* plant communities decreased with increasing grazing intensity (Wang et al., 2023). Meanwhile, *S. breviflora* can also enhance the ability of tolerating grazing through some modes. It has been shown that grazing significantly increased the cover, density, and proportion in standing crop of *S. breviflora* but decreased the height. The spatial distribution of *S. breviflora* was strongly dependent upon the sampling unit and grazing intensity, implying that spatial aggregation was beneficial to the survival of this dominant species under heavy grazing (Lv et al., 2019). Majority of research about changing of *S. breviflora* under different intensities were focused on biomass and spatial aggregation. However, very little is known about the morphological structure and transcriptional regulation response to different grazing intensities in *S. breviflora*. Therefore, transcriptome and anatomical analyses of *S. breviflora* under different grazing intensities, including no grazing (CK), moderate grazing (MG), and heavy grazing (HG), were performed to explore the effect of different grazing intensities on the anatomical structure and genes of *S. breviflora* in desert steppe.

## 2 Materials and methods

### 2.1 General situation of test area and design of experiments

The study area located in the Siziwang base of Comprehensive Experimental Demonstration Center of Inner Mongolia Autonomous Region Academy of Agricultural and Animal Husbandry Sciences (41°47′17″ N, 111°53′46″ E, altitude 1,450 m). The average precipitation per year is 220 mm. The soil is light chestnut calcium soil, which is desert steppe with a relatively simple composition of plant species and a low grass cover, whose grass type is *Stipa breviflora*, *Artemisia frigida*, and *Cleistogenes songorica*. The experiment was conducted in a randomized block design, and 50 hm^2^ of natural grassland with flat terrain and uniform vegetation was selected for enclosure and divided into nine plots, with the area of each plot basically the same as that of the other, which was 5.6 hm^2^. The nine plots were grouped into three blocks, with three treatments in each block, which were heavy grazing (HG), moderate grazing (MG), and no grazing (CK) (**Supplementary Figure S1**). The heavy grazing (2.71 sheep · hm^-2^ · half a year), moderate grazing (1.82 sheep · hm^-2^ · half a year), and no grazing (0 sheep · hm^-2^ · half a year) treatments were separated by fences. Local adult 2-year-old Capricorn sheep were used in the experiment, and the grazing period was from June to November every year, with grazing at 6:00 a.m. every day and returning to grazing at 6:00 p.m. in the evening. The grazing site was established in 2004. A total of 17 years of enclosure grazing experiments has been conducted from 2004 to the present. Sampling was done in mid-August (peak growth period).

### 2.2 Observations on the anatomical structure of *S. breviflora* leaves under different grazing intensities

In each plot of the study area, randomly selected *S. breviflora* leaves with uniform growth were put into FAA fixative and fixed in a refrigerator at 4°C for 1 week, and then sections were made according to the methods and steps of paraffin sectioning, followed by observation and imaging under a ×40 microscope. Determination of cell wall thickness was done using a scale in a microscope.

### 2.3 RNA extraction, RNA sequencing, and gene expression analysis

Leaves were collected from test plots directly and then put into liquid nitrogen for RNA-seq, and the rest of the samples were placed in a refrigerator at -80°C for storage. RNA-seq was conducted without reference transcriptome. Total RNA was extracted from 18 samples using Trizol reagent (Invitrogen, USA), RNA quality and concentration were checked using NanoDrop, and the integrity was checked using 1% agarose gel electrophoresis. A total of 18 cDNA libraries were constructed using 1 μg RNA from each sample, following the EasyQuick RT MasterMix (*CWBIO*) protocol. The prepared cDNA libraries were sequenced on Illumina HiSeq2000 platform. 2×MagicSYBR Mixture (1×) (*CWBIO*) was employed for quantitative real-time PCR (qRT-PCR). qRT-PCR was conducted by ABI 7500 Fast Applied Biosystem (Thermo Fisher). The qPCR results were calculated using the 2^-ΔΔCT^ method (Livak and Schmittgen, 2001). The primers used for qRT-PCR are listed in **Supplementary Table S1**.

The expression levels of each gene were quantified by normalizing the total gene counts with the effective library size. The FPKM expression levels and counts for all unigenes were estimated in each replicate by RSEM (Li and Dewey, 2011). Gene function was annotated based on the following databases: NR (NCBI non-redundant protein sequences), Pfam (protein family), KOG/COG/eggNOG (clusters of orthologous groups of proteins), Swiss-Prot (a manually annotated and reviewed protein sequence database), KEGG (Kyoto Encyclopedia of Genes and Genomes), and GO (Gene Ontology).

### 2.4 Functional analysis differentially expressed genes

Differentially expressed genes (DEGs) were determined by DEseq2 (Love et al., 2014) (fold change ≥2, FDR < 0.01), and unigenes with a significant *p*-value were likewise identified (<0.05). The annotated DEGs were used in functional enrichment analysis. Kyoto Encyclopedia of Genes and Genomes (KEGG) pathway enrichment analysis and GO category annotation of significant DEGs were performed by clusterProfler with adjusted *p*-value.

### 2.5 Determination of cell wall composition

We determined the composition of cell wall, which included lignin, cellulose, hemicellulose, and pectin, using the Lignin Content Assay Kit (AD9878, ADANTI, Wuhan), Cellulose Content Assay Kit (AD9867, ADANTI, Wuhan), Hemicellulose Content Assay Kit (AD9869, ADANTI, Wuhan), and Pectin Content Assay Kit (AD9864, ADANTI, Wuhan) separately. All kits from ADANTI and all experimental procedures were performed in accordance with the manufacturer’s instructions.

### 2.6 ATP synthase and Rubisco-activating enzyme assays

Samples were collected from test plots directly and then put into liquid nitrogen to activate enzyme activity assays, and the rest of the samples were placed in a refrigerator -80°C for storage. Determined by using a plant ATP synthase enzyme-linked immunoassay kit (AD9033, ADANTI, Wuhan) and plant Rubisco-activating enzyme (RCA) ELISA Kit (AD9158, ADANTI, Wuhan), the absorbance (OD value) was measured at 450 nm with a microplate reader, and the concentration of Rubisco-activating enzyme (RCA) and ATP synthase in the samples was calculated by the standard curve. All experimental procedures were performed in accordance with the manufacturer’s instructions.

### 2.7 Statistic analysis

Data were collected using Microsoft Excel 2013 and were presented as mean ± standard deviation. Graphpad Prism 9 was used to perform *t*-tests, and the *p*-value was calculated. Graphpad Prism 9 and Adobe Illustrator 2022 were used to construct the graphs.

## 3 Results

### 3.1 Response of leaf anatomical structure to different grazing intensities

**Figure 1:**
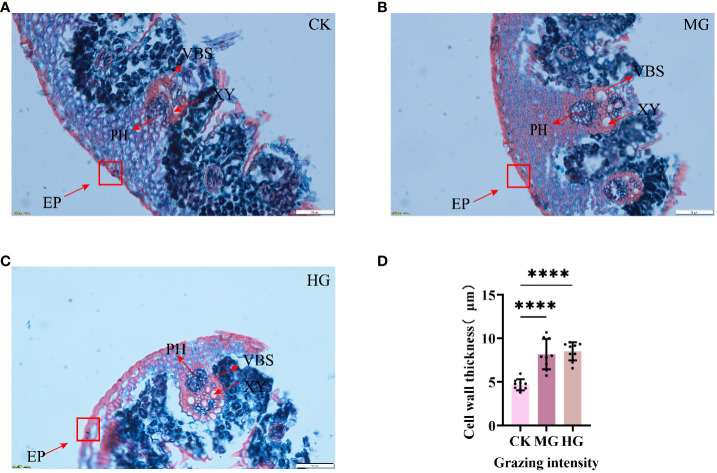
Structure of leaves under different grazing intensities. **(A)** Structure of leaves under no grazing. **(B)** Structure of leaves under moderate grazing. **(C)** Structure of leaves under heavy grazing. **(D)** Cell wall thickness of leaves. EP, epidermis; XY, xylem; PH, phloem; VBS, vascular bundle sheath. **** indicates P<0.0001.

To explore the response of *S. breviflora* anatomical structure to different grazing intensities, a paraffin section has been conducted. The results showed that the area of xylems was increased clearly, suggesting that grazing results in increasing lignification. Furthermore, we found that the epidermis cell significantly thickens with grazing intensity (**Figures 1A–C**), which increased about two times (**Figure 1D**). Taken together, these findings suggest that a thickened epidermis cell wall and increased xylems belonged to a kind of defense strategy, which can avoid leaf crumpling caused by droughts after grazing.

### 3.2 Transcriptome analysis of *S. breviflora*

To further explore the mechanism of *S. breviflora* response to grazing, RNA-seq has been executed. A total of 124.49-Gb clean data was obtained after the elimination of low-quality reads, and the clean data of each sample reached 5.91 Gb, with Q30 above 92.79% (**Supplementary Table S2**). Additionally, a total of 38,302 unigenes were obtained after assembly, and the N50 of unigene was 1,204, among which 10,136 unigenes with length above 1 kb were obtained (**Supplementary Table S3**).

**Figure 2:**
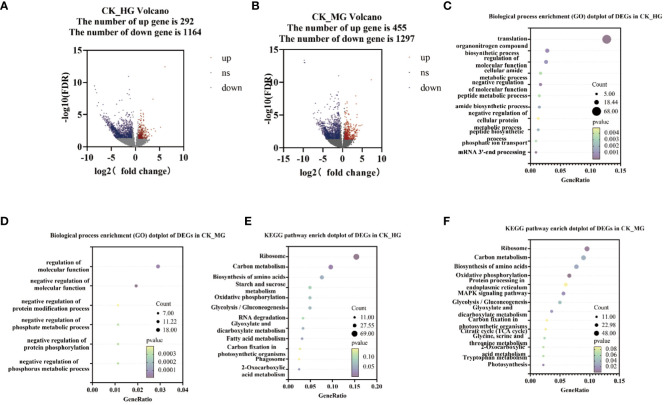
Number and annotations of “differentially expressed genes” (DEGs) from comparisons CK_HG and CK_MG. **(A)** Number of DEGs in CK_HG. **(B)** Number of DEGs in CK_MG. **(C)** Biological process of GO enrichment of differentially expressed genes in CK_HG. **(D)** Biological process of GO enrichment of differentially expressed genes in CK_MG. **(E)** KEGG pathway enrichment of differentially expressed genes in CK_HG. **(F)** KEGG pathway enrichment of differentially expressed genes in CK_MG. Red dots indicate up regulated genes; blue dots indicate down regulated genes.

To explore the genes’ response to different grazing intensities, DEGs analysis was performed among the three groups compared: CK_HG, CK_MG, and HG_MG. The results showed that 1,456 genes were found to differentiate between CK and HG, of which 292 were upregulated DEGs and 1,164 were downregulated DEGs (**Figure 2A**). Furthermore, 1,752 genes were found to differentiate between CK and MG, of which 455 were upregulated DEGs and 1,297 were downregulated DEGs (**Figure 2B**). KEGG enrichment analysis and Gene Ontology (GO) enrichment analysis were performed on these DEGs. The results of the biological process enrichment (GO) showed that most of the DEGs in CK_HG were involved in “translation”, “organonitrogen compound biosynthetic process”, “regulation of molecular function”, “cellular amide metabolic process”, “negative regulation of molecular function”, and “peptide metabolic process” (**Figure 2C**). In the group of CK_MG, the results of the biological process showed that most of the DEGs were involved in “regulation of molecular function”, “negative regulation of molecular function”, “negative regulation of protein modification process”, “negative regulation of phosphate metabolic process”, “cellular component assembly involved in morphogenesis”, and “negative regulation of phosphorus metabolic process” (**Figure 2D**).

In the group of CK_HG, there were 449 DEGs annotated in the KEGG pathway. The results of the KEGG enrichment showed that there were several pathways enriched in KEGG. Among these pathways, “glycolysis/gluconeogenesis” (22 DEGs), “oxidative phosphorylation” (22 DEGs), “starch and sucrose metabolism” (22 DEGs), “biosynthesis of amino acids” (34 DEGs), “ribosome” (69 DEGs), and “carbon metabolism” (43 DEGs) were enriched with a large number of DEGs compared to other pathways (**Figure 2E**). In the group of CK_MG, there were 503 DEGs annotated in the KEGG pathway. The results of the KEGG enrichment showed that there were several pathways enriched in KEGG. Among these pathways, “glycolysis/gluconeogenesis” (25 DEGs), “MAPK signaling pathway – plant” (28 DEGs), “oxidative phosphorylation” (33 DEGs), “protein processing in endoplasmic reticulum” (30 DEGs), “biosynthesis of amino acids” (39 DEGs), “ribosome” (48 DEGs), and carbon metabolism (45 DEGs) were enriched with many DEGs compared to other pathways (**Figure 2F**).

These results suggested that the exposure of *S. breviflora* to the long-term grazing ecological conditions in the desert grassland has given rise to important adjustments in its gene expression patterns, which were associated with multiple pathways, such as energy metabolism, carbon metabolism, and biosynthesis of amino acids. These changes could support the long-term survival of *S. breviflora* in the desert and in high grazing environments, and changes may be the result of the ecological domestication of the species.

### 3.3 Key pathways’ response to different grazing intensities

#### 3.3.1 Glycolysis/gluconeogenesis pathway

**Figure 3:**
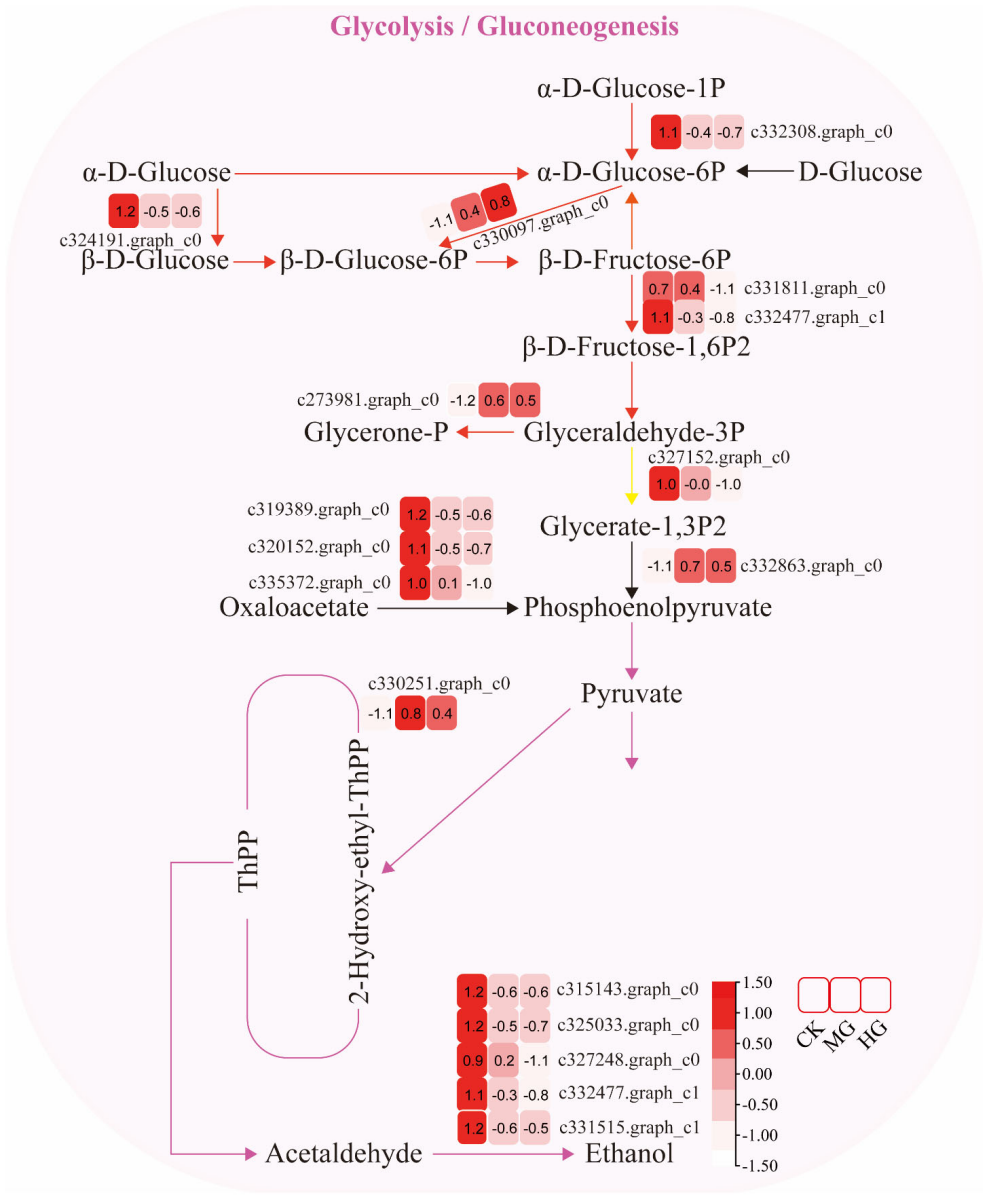
Glycolysis/gluconeogenesis pathway and differentially expressed genes. Boxes indicate the expression of differential genes under different treatments within the pathway. Shades of color indicate a high or low gene expression; darker colors indicate a higher gene expression.

The expression of genes encoding enzymes involved in “glycolysis/gluconeogenesis” changed significantly under moderate and heavy grazing conditions. There were 17 DEGs encoding key enzymes and proteins in the “glycolysis/gluconeogenesis” metabolic pathways, of which four DEGs were upregulated and 13 DEGs were downregulated. The upregulated genes code many enzymes, including pyruvate dehydrogenase E1 component subunit beta-3, putative glucose-6-phosphate 1-epimerase, elicitor-responsive protein 1, and enolase. These enzymes were working in the chloroplasts and mitochondria, indicating that genes related to photosynthesis and respiration were activated under moderate and heavy grazing. The downregulated genes code many enzymes, including glyceraldehyde 3-phosphate dehydrogenase, phosphoenolpyruvate carboxykinase, phosphofructokinase, alcohol dehydrogenase, aldose 1-epimerase, phosphoglucomutase/phosphomannomutase, aldehyde dehydrogenase family, Aldo/keto reductase family, and putative esterase. Therefore, we supposed that these up/downregulated genes play a central role in plant energy homeostasis and adjust metabolism to the response grazing through glycolysis/gluconeogenesis and a central role in the tricarboxylic acid cycle (**Figure 3**).

#### 3.3.2 Starch and sucrose metabolism pathway

**Figure 4:**
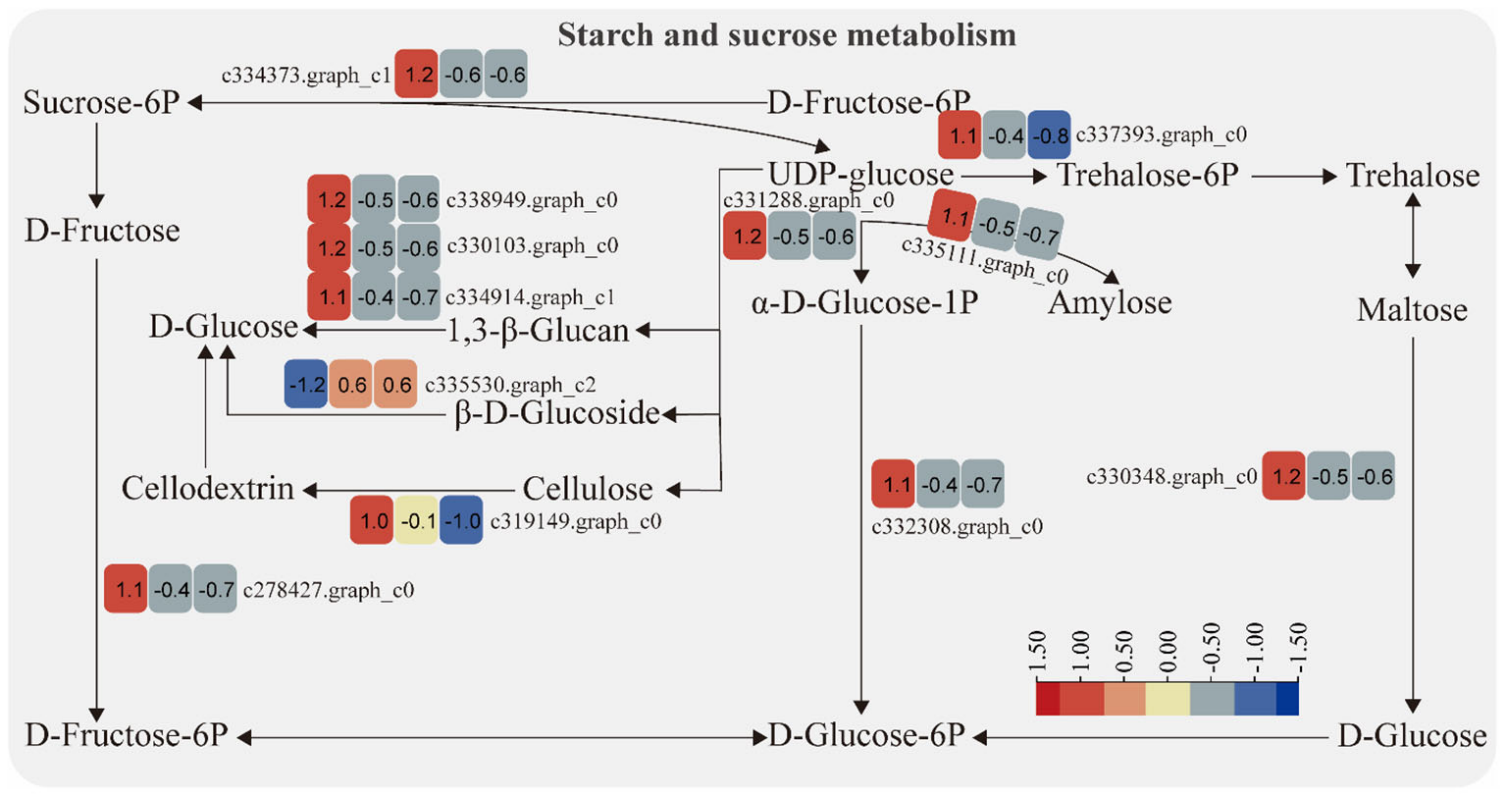
Starch and sucrose metabolism pathway and differentially expressed genes. Boxes indicate the expression of differential genes under different treatments within the pathway. Shades of color indicate a high or low gene expression; darker colors indicate a higher gene expression.

The expression of genes encoding enzymes involved in “starch and sucrose metabolism” changed significantly under moderate and heavy grazing conditions. There were 12 DEGs encoding key enzymes and proteins in the “starch and sucrose metabolism” metabolic pathways, of which one DEG was upregulated and 11 DEGs were downregulated. This upregulated genes coding beta-glucosidase 7, which engaged in plant cell wall formation and degradation. Therefore, we supposed that this gene upregulated under moderate and heavy grazing to thicken the cell walls. These downregulated genes coding type I phosphodiesterase/nucleotide pyrophosphatase, glycogen synthase, cellulase, glycosyltransferase, sucrose-6F-phosphate phosphohydrolase, sucrose synthase, phosphoglucomutase/phosphomannomutase, and glycosyl hydrolase (**Figure 4**).

#### 3.3.3 Oxidative phosphorylation pathway

**Figure 5:**
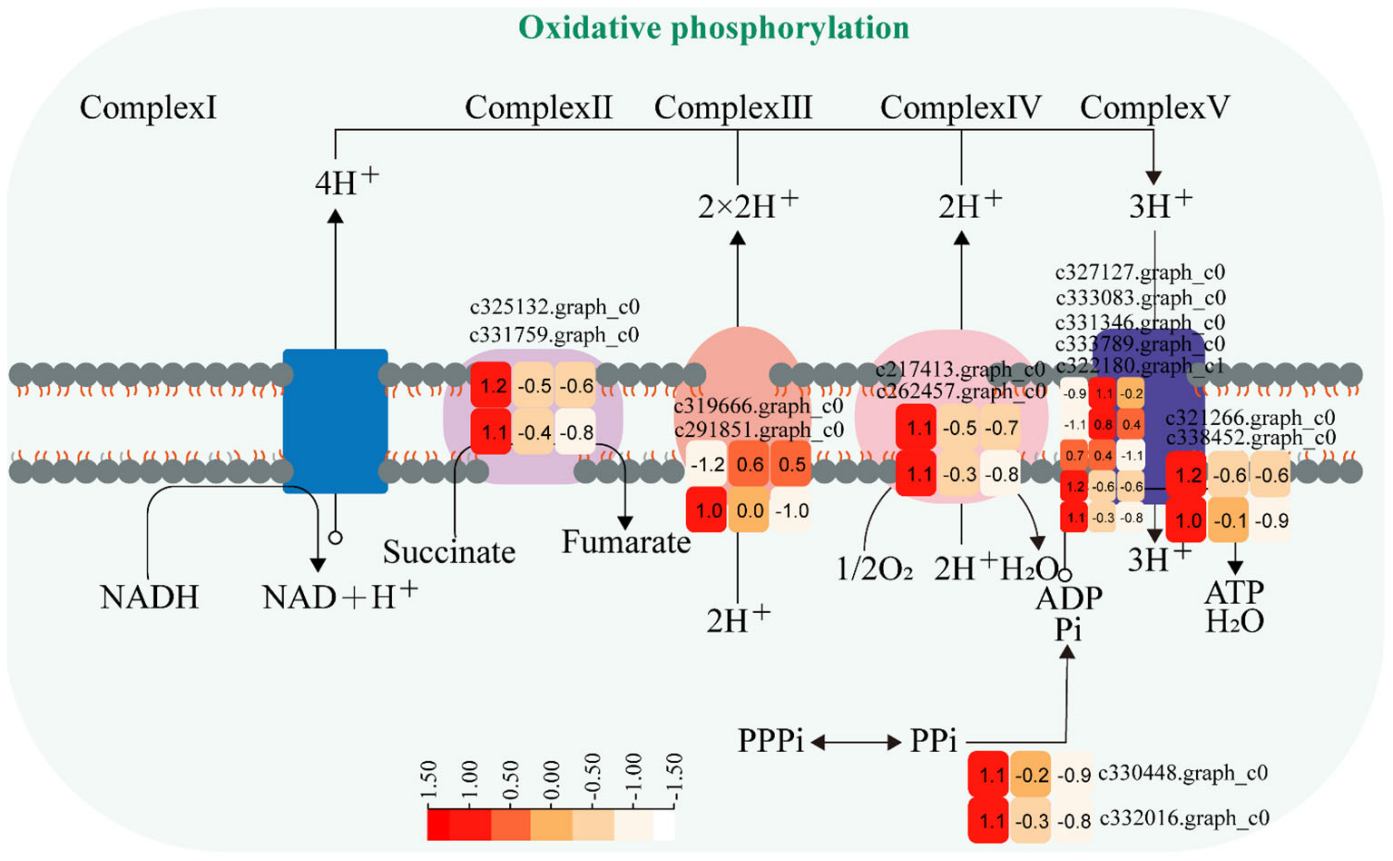
Oxidative phosphorylation pathway and differentially expressed genes. Boxes indicate the expression of differential genes under different treatments within the pathway. Shades of color indicate a high or low gene expression; darker colors indicate a higher gene expression.

The expression of genes encoding enzymes involved in “oxidative phosphorylation” changed significantly under moderate and heavy grazing conditions. There were 15 DEGs encoding key enzymes and proteins in the “oxidative phosphorylation” metabolic pathways, of which three DEGs were upregulated and 12 DEGs were downregulated. The upregulated genes code many enzymes and proteins, including ATP synthase protein, cytochrome b-c1 complex subunit, and V-type proton ATPase subunit F. The downregulated genes code many enzymes and proteins, including E1–E2 ATPase, inorganic pyrophosphatase, ubiquitin-conjugating enzyme, inorganic pyrophosphatase, cytochrome b (C-terminal)/b6/petD, ATP synthase subunit C, FAD binding domain, ATP synthase A chain, cytochrome c oxidase subunit III, and ATP synthase alpha/beta family. These proteins were working in chloroplasts and mitochondria, indicating that genes also related to photosynthesis and respiration were activated under moderate and heavy grazing. Therefore, we supposed that these up/downregulated genes play a central part in plant energy homeostasis and adjust metabolism to response grazing through oxidative phosphorylation (**Figure 5**).

#### 3.3.4 Carbon metabolism pathway

**Figure 6:**
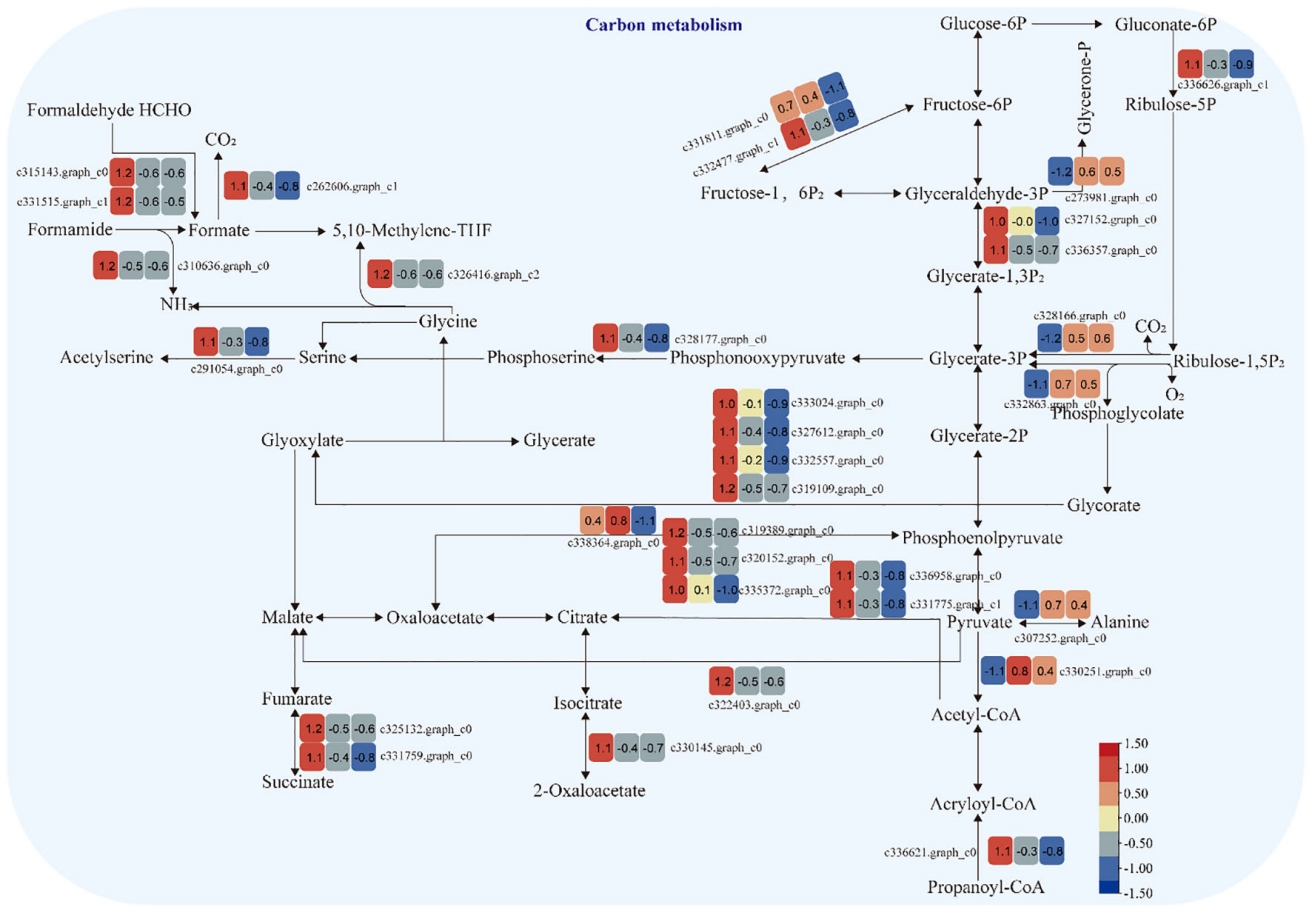
Carbon metabolism pathway and differentially expressed genes. Boxes indicate the expression of differential genes under different treatments within the pathway. Shades of color indicate a high or low gene expression; darker colors indicate a higher gene expression.

The expression of genes encoding enzymes involved in “carbon metabolism” changed significantly under moderate and heavy grazing conditions. There were 32 DEGs encoding key enzymes and proteins in the “carbon metabolism” pathways, of which five DEGs were upregulated and 27 DEGs were downregulated. These upregulated genes code pyruvate dehydrogenase E1 component subunit beta-3, glutamate–glyoxylate aminotransferase 2, ribulose bisphosphate carboxylase small chain A, elicitor-responsive protein 1, and enolase. These downregulated genes code glyceraldehyde 3-phosphate dehydrogenase, isopropylmalate dehydrogenase, catalase, malic enzyme, phosphoenolpyruvate carboxykinase, citrate synthase, phosphofructokinase, phosphoenolpyruvate carboxykinase (ATP), 6-phosphogluconate dehydrogenase, D-isomer-specific 2-hydroxyacid dehydrogenase, alcohol dehydrogenase, PPR repeat family, phosphofructokinase, serine acetyltransferase, putative esterase, Acyl-CoA oxidase, and Acyl-CoA dehydrogenase. These up/downregulated genes engaged in glycolysis, the tricarboxylic acid cycle during respiration. “Ribulose bisphosphate carboxylase small chain A”, which is a key enzyme in photosynthesis, especially determines the rate of carbon assimilation and plant photorespiration (**Figure 6**).

### 3.4 qRT-PCR of DEGs in key pathway

**Figure 7:**
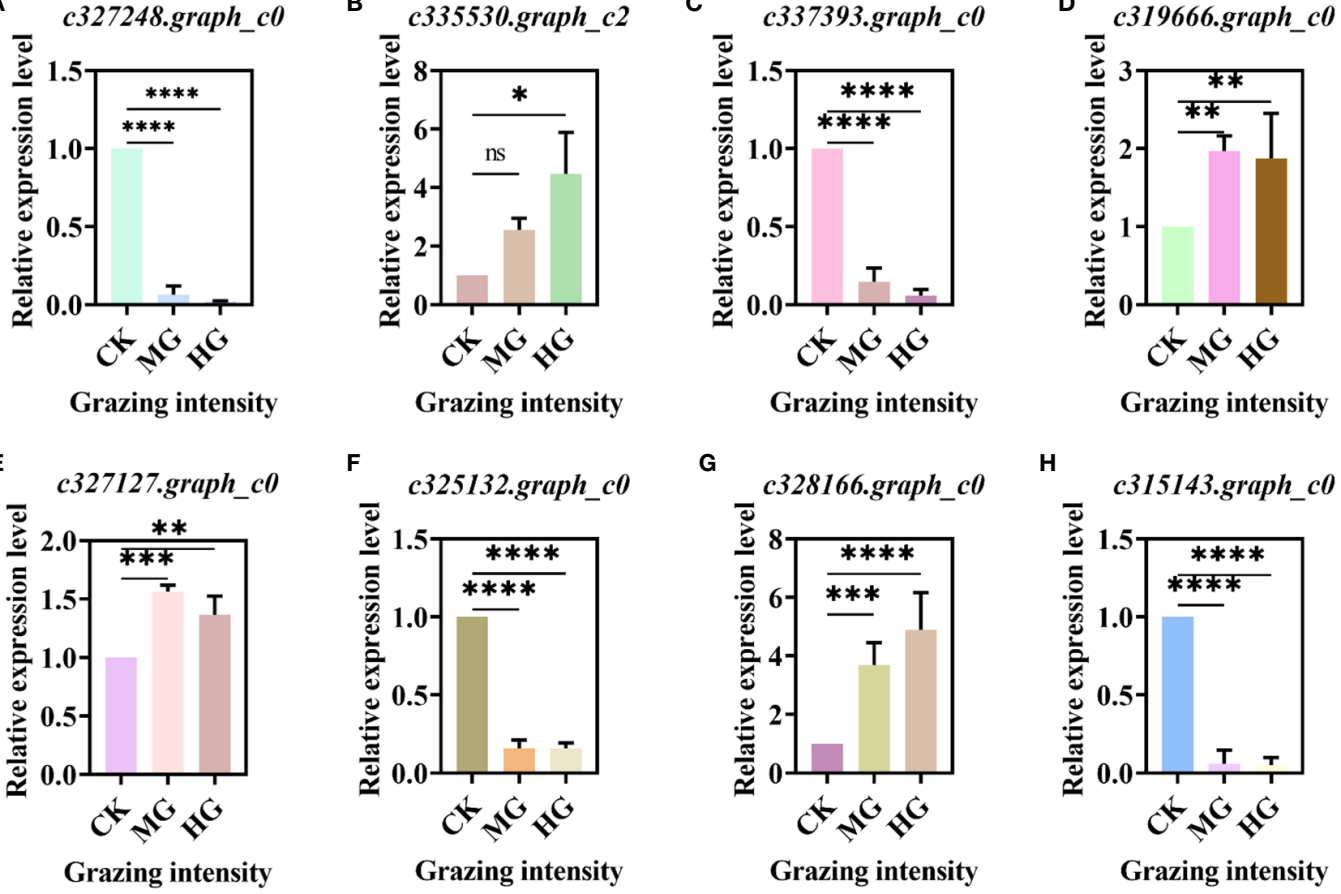
Relative expression level of differentially expressed genes (DEGs) in key pathway. **(A)** DEGs in glycolysis/gluconeogenesis pathway. **(B, C)** DEGs in starch and sucrose metabolism pathway. **(D, E)** DEGs in oxidative phosphorylation pathway. **(F–H)** DEGs in carbon metabolism pathway. * indicates P<0.05, ** indicates P<0.01,*** indicates P<0.001, **** indicates P<0.0001, ns indicates no significance.

We selected some DEGs from key pathways randomly and verified these genes by qRT-PCR. The results showed that the gene in the glycolysis/gluconeogenesis pathway was downregulated significantly (**Figure 7A**). The gene “c335530.graph_c2” in the starch and sucrose metabolism pathway was upregulated significantly (**Figure 7B**), while the gene “c337393.graph_c0” was downregulated significantly (**Figure 7C**). DEGs “c319666.graph_c0” and “c327127.graph_c0” in the oxidative phosphorylation pathway were all upregulated significantly compared to CK (**Figures 7D, E**). Genes “c325132.graph_c0” and “c315143.graph_c0” in the carbon metabolism pathway were downregulated significantly (**Figures 7F, G**), while the gene “c328166.graph_c0” was upregulated significantly (**Figure 7H**). The trends in all genes were consistent with the transcriptome.

### 3.5 Cell wall composition and activities of ATP synthase and Rubisco-activating enzyme increased significantly after grazing

**Figure 8:**
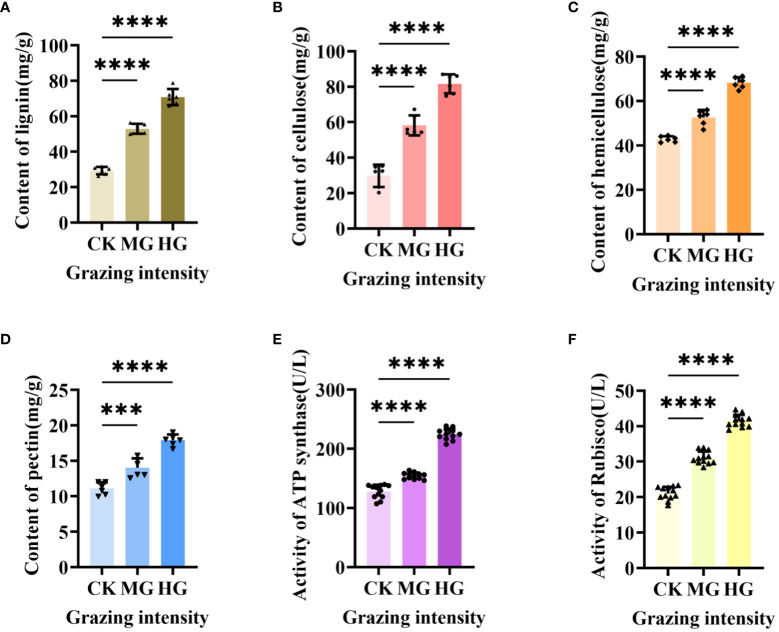
Cell wall composition and activity of two enzymes under different grazing intensities. **(A)** Content of lignin. **(B)** Content of cellulose. **(C)** Content of hemicellulose. **(D)** Content of pectin. **(E)** Activity of ATP synthase. **(F)** Activity of Rubisco-activating enzyme. *** indicates P<0.001, **** indicates P<0.0001.

Our previous results showed that the upregulated gene in starch and sucrose metabolism engaged in plant cell wall formation and degradation by annotation, but little is known about the exact function of this gene in regulating cell wall. Therefore, we determined the composition of cell wall, which included lignin (**Figure 8A**), cellulose (**Figure 8B**), hemicellulose (**Figure 8C**), and pectin (**Figure 8D**). The results showed that the content level of the four compositions was increased dramatically and significantly under moderate grazing and heavy grazing, which, together with the results above, demonstrated that over-grazing brings about an increase in the content of the cell wall composition and, in turn, leads to a thickened cell wall. Furthermore, we proposed that the gene plays a key role in responding to over-grazing.

Previous results showed that the differentially expressed genes in glycolysis/gluconeogenesis metabolism, energy metabolism, and carbon metabolism were focused on photosynthesis and respiration reaction. These two reactions usually related to many enzymes. To explore how enzymes work under different grazing intensities, we determined the activity of ATP synthase (**Figure 8E**) and Rubisco-activating enzyme (RCA) (**Figure 8F**). From the results, the activities of ATP synthase and Rubisco activating enzyme increased significantly with enhanced grazing intensity and differed significantly between moderate and heavy grazing intensities.

## 4 Discussion

In the current study, over-grazing suppressed plant growth, reduced the soil quality, and is closely associated with grassland degradation. Leaves are important organs in the growth and development of individual plants. During the evolutionary process of plants, the leaves were relatively sensitive and plastic to environmental changes, and its structural characteristics best reflect the plant’s adaptation to the environment (Henry and Aarssen, 1997). However, there were few research on the response of the leaf structure to different grazing intensities. As the main organ is consumed by grazing, the leaves of *S. breviflora* were significantly influenced by herbivory. Our results showed that epidermis cell thickness and xylems were significantly increased with grazing intensity. Previous studies have indicated that *Kobresia humilis* leaf tips showed thickening of the proximal–axial cuticle under both moderate and heavy grazing compared to the no-grazing treatment and reached significant levels under heavy grazing, and the distal–axial cuticle thickness at the leaf bases increased progressively with the gradual intensification of grazing intensity (Xiao et al., 2023). Together with previous studies, the results demonstrate the central grazing avoidance strategy played by *S. breviflora* in response to grazing disturbance, or it may be due to a certain degree of degradation of the grassland under overgrazing, resulting in an increase in the bare surface area and accelerated evaporation of soil moisture, and *S. breviflora* showed thickening of epidermal cells in response to the water-deficient environment.

Gene expression is the proximate mechanism that links genotype to phenotype and plays a central role in cellular adaptation to environmental changes (Kenkel and Matz, 2016). In our study, several genes were downregulated for expression, and a small number of genes were upregulated for expression. Most of the differentially expressed genes are enriched to the glycolysis/gluconeogenesis metabolism pathway, starch and sucrose metabolism pathway, energy metabolism pathway, and carbon metabolism pathway. Overall, these findings are in accordance with findings reported by Wang et al. (2022). In the process of plant growth and development, carbohydrates were employed for photosynthesis and respiration, providing a carbon framework and energy for plant growth and metabolism and strengthening plant resistance to stress (Kou et al., 2021). In the “glycolysis/gluconeogenesis” pathways, the upregulated genes code many enzymes, including pyruvate dehydrogenase E1 component subunit beta-3, putative glucose-6-phosphate 1-epimerase, elicitor-responsive protein 1, and enolase. The downregulated genes code many enzymes, including glyceraldehyde 3-phosphate dehydrogenase, phosphoenolpyruvate carboxykinase, phosphofructokinase, alcohol dehydrogenase, aldose 1-epimerase, phosphoglucomutase/phosphomannomutase, aldehyde dehydrogenase family, Aldo/keto reductase family, and putative esterase. There were two pathways that interacted and regulated the carbohydrate metabolism of *S. breviflora* to adapt to external environment changes. In the “starch and sucrose metabolism” pathway, there was only one upregulated gene which codes beta-glucosidase 7 that is engaged in plant cell wall formation and degradation. Therefore, we supposed that this gene upregulated under moderate and heavy grazing to thicken the epidermis cell walls. Moreover, the results showed that the content of the compositions was increased dramatically and significantly under the moderate and heavy grazing. This implies that over-grazing brings about the content of cell wall compositions of epidermis being increased, which, in turn, leads to epidermis cell wall thickening. Furthermore, we proposed that the upregulated gene will facilitate cell wall formation and thickening. In the “oxidative phosphorylation” pathway, there were three DEGs upregulated, and the upregulated genes code many enzymes and proteins including ATP synthase protein, cytochrome b-c1 complex subunit, and V-type proton ATPase subunit F. Then, we determined the activity of ATP synthase. The results showed that the activities of ATP synthase increased significantly with enhanced grazing intensity and differed significantly between moderate and heavy grazing intensities. In the “carbon metabolism” pathway, there were five DEGs upregulated, and these upregulated genes code pyruvate dehydrogenase E1 component subunit beta-3, glutamate–glyoxylate aminotransferase 2, ribulose bisphosphate carboxylase small chain A, elicitor-responsive protein 1, and enolase, which engaged in glycolysis, the tricarboxylic acid cycle during respiration. “Ribulose bisphosphate carboxylase small chain A”, which is a key enzyme in photosynthesis, especially determines the rate of carbon assimilation and plant photorespiration. Then, we determined the activity of Rubisco-activating enzyme (RCA). From the results, the activities of Rubisco-activating enzyme increased significantly with enhanced grazing intensity and differed significantly between moderate and heavy grazing intensities.

Livestock grazing directly affects the absorption and utilization of solar energy by plant morphology changes in response to grazing. The ability of the plant to use light is enhanced due to the reduction in leaves after grazing, i.e., decreased shading. However, on the other hand, the decreased leaf area may limit their ability to obtain sufficient light (Stagnari et al., 2018). Different grazing stresses also affected the physiological responses of plants, such as compensatory growth and changes in photosynthetic capacity (Rivas et al., 2017). The effect of grazing on leaf photosynthesis has been extensively studied. There was a study that investigated the impact of different grazing intensities [light grazing (LG), medium grazing (MG), and heavy grazing (HG)] on leaf photosynthesis parameters and photosynthetic pigments of three grass species on an alpine steppe in the Qilian Mountains. Grazing had a positive effect on the leaf photosynthesis parameters of *S. purpurea* and *L. secalinus* (Li et al., 2021). However, it is not clear how grazing regulates photosynthesis and light compensation. In fact, the primary response to removal of tissues by herbivory is regrowth through the reconstruction of damaged tissues and organs, which is achieved by increased CO_2_ assimilation capacity (Lu et al., 2017). Ribulose-1,5-bisphosphate carboxylase/oxygenase (Rubisco) is a key enzyme in the carbon reactions of photosynthesis (Wang et al., 2023). In our study, the activities of the Rubisco-activating enzyme increased significantly with enhanced grazing intensity. Rich RCA accelerates CO_2_ fixation and activates Rubisco (Dang et al., 2021). Highly efficient photosynthetic CO_2_ fixation depends not only on the carboxylation capacity of Rubisco but also on the regeneration of RuBP (Raines, 2011). This suggests that the enzyme was actively engaged in maintaining photosynthesis under grazing stress. The significant upregulation of “ribulose bisphosphate carboxylase small chain A” unigene expression might be highly related to the hyper-compensatory photosynthesis of *Stipa breviflora*, especially under LG and MG conditions. Moreover, chloroplast ATP synthase produces the ATP needed for photosynthesis and plant growth (Yamamoto et al., 2023). In our study, the activities of ATP synthase increased significantly with enhanced grazing intensity. This is also a potentially beneficial consequence for photosynthesis and plant regrowth after grazing.

## 5 Conclusions

Our research found that epidermis cells and xylems significantly thicken with grazing intensity. Furthermore, the components of the cell wall, such as lignin, cellulose, hemicellulose, and pectin, were all increased dramatically and significantly under both moderate and heavy grazing. Transcriptome analysis showed that the differentially expressed genes related to different grazing intensities were also engaged in plant cell wall formation and photosynthesis and respiration. In addition, the activities of ATP synthase and Rubisco-activating enzyme (RCA) increased significantly with enhanced grazing intensity and differed significantly between moderate and heavy grazing intensities. Taken together, *Stipa breviflora* has evolved a grazing tolerance strategy under long-term grazing conditions, influencing photosynthesis and respiration in terms of its own structure and enzyme activities in the body, to maintain normal life activities under different grazing conditions.

## Data availability statement

The data presented in the study are deposited in the SRA repository, accession number PRJNA1071026.

## Author contributions

XW: Writing – original draft, Visualization, Validation, Software, Formal analysis, Data curation. JW: Writing – review & editing, Validation, Investigation. RD: Writing – review & editing, Validation, Investigation. ZZ: Writing – review & editing, Methodology, Conceptualization. YW: Writing – review & editing, Resources, Funding acquisition, Conceptualization. FM: Writing – review & editing, Supervision, Resources.
