# Supplementary material for: Transcriptome and anatomical analysis of Stipa breviflora in response to different grazing intensities in desert steppe
Source: Front Plant Sci. 2024 Jun 10;15:1414093. doi: 10.3389/fpls.2024.1414093 (PMC11194427; doi:10.3389/fpls.2024.1414093)
Supplement: Supplementary file 1 [file DataSheet_1.docx]

**Supplemental figure: Figure S1_** **Grazing experimental design. CK: no grazing, MG: moderate grazing, HG: heavy grazing.**

**
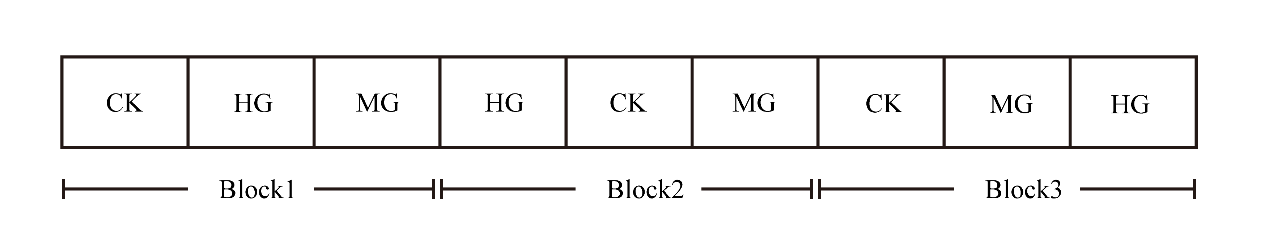
**

**Supplemental table: Table S1_** **Primers of qRT-PCR**

| **Name of primers** | **Sequence of primers (5’ – 3’)** |
| --- | --- |
| c335530.graph_c2-F | ACTAGGCACTAGCCAATCGC |
| c335530.graph_c2-R | CAGCTAGGCACGCCGTATAG |
| c337393.graph_c0-F | GGTGCGGAATATCTCCGAGG |
| c337393.graph_c0-R | TGGATCCACGACTACCACCT |
| c327248.graph_c0-F | ATCTGTCCACACACGCCAAT |
| c327248.graph_c0-R | CTATGGCCAAGGGTGACGTT |
| c332863.graph_c0-F | AAGAATGAGTGGGGCTGGTG |
| c332863.graph_c0-R | TTACCAGCAAGGTTGGCGAT |
| c319666.graph_c0-F | CTGGCTGCCGAAAAACTGTC |
| c319666.graph_c0-R | TCATATCACTTGCGCACCCT |
| c327127.graph_c0-F | GGTCTCAGTGTTGCCGCTAT |
| c327127.graph_c0-R | CCAGAAGATGGTCAGGAGCG |
| c325132.graph_c0-F | GCTTGTGCAAATAGGGTGGC |
| c325132.graph_c0-R | TCGTTGCATGTTGAGTCGGA |
| c328166.graph_c0-F | CAGTGCATCAGCTTCATCGC |
| c328166.graph_c0-R | GTGACCTGTTGGGTTCGACT |
| c315143.graph_c0-F | GTAGAAGCATTCGCCGCAAG |
| c315143.graph_c0-R | CCTCAAGATCACAGGCAGCA |
| TLF-F | CCCTCAGTGTGTGTTTGACC |
| TLF-R | CTTGAGACCCTTCCTCTTGC |

**Supplemental table: Table S2_** **Statistics of the RNA-Seq data**

| **Samples** | **Clean reads** | **Q30(%)** | **GC%** |
| --- | --- | --- | --- |
| CK-1 | 29,220,073 | 92.79% | 55.08% |
| CK-2 | 21,988,951 | 93.32% | 47.48% |
| CK-3 | 36,391,046 | 93.17% | 54.73% |
| CK-4 | 24,331,615 | 94.25% | 55.84% |
| CK-5 | 21,311,264 | 93.31% | 54.98% |
| CK-6 | 21,212,611 | 93.95% | 54.59% |
| HG-1 | 24,472,034 | 94.36% | 54.33% |
| HG-2 | 23,575,314 | 93.90% | 55.63% |
| HG-3 | 22,140,079 | 94.00% | 54.48% |
| HG-4 | 21,588,223 | 94.40% | 54.06% |
| HG-5 | 21,140,872 | 94.60% | 55.00% |
| HG-6 | 21,479,297 | 94.25% | 54.71% |
| MG-1 | 22,821,916 | 93.87% | 54.36% |
| MG-2 | 22,063,965 | 94.32% | 54.38% |
| MG-3 | 20,789,385 | 94.51% | 54.22% |
| MG-4 | 21,140,644 | 93.93% | 54.88% |
| MG-5 | 19,818,795 | 94.23% | 54.15% |
| MG-6 | 21,565,523 | 94.46% | 54.67% |

**Supplemental table: Table S3_Statistical table of assembly result**

| **Length Range** | **Transcript** | **Unigene** |
| --- | --- | --- |
| 300-500 | 19,105(34.85%) | 16,642(43.45%) |
| 500-1000 | 16,960(30.93%) | 11,523(30.08%) |
| 1000-2000 | 12,794(23.33%) | 6,912(18.05%) |
| 2000+ | 5,968(10.88%) | 3,224(8.42%) |
| Total Number | 54,828 | 38,302 |
| Total Length | 54,956,568 | 33,716,460 |
| N50 Length | 1,389 | 1,204 |
| Mean Length | 1002.34 | 880.28 |
